# Typing Late Prehistoric Cows and Bulls—Osteology and Genetics of Cattle at the Eketorp Ringfort on the Öland Island in Sweden

**DOI:** 10.1371/journal.pone.0020748

**Published:** 2011-06-22

**Authors:** Ylva Telldahl, Emma Svensson, Anders Götherström, Jan Storå

**Affiliations:** 1 Osteoarchaeological Research Laboratory, Department of Archaeology and Classical Studies, Stockholm University, Stockholm, Sweden; 2 Department of Evolutionary Biology, Uppsala Universitet, Uppsala, Sweden; Ecole Normale Supérieure de Lyon, France

## Abstract

Human management of livestock and the presence of different breeds have been discussed in archaeozoology and animal breeding. Traditionally osteometrics has been the main tool in addressing these questions. We combine osteometrics with molecular sex identifications of 104 of 340 morphometrically analysed bones in order to investigate the use of cattle at the Eketorp ringfort on the Öland island in Sweden. The fort is dated to 300–1220/50 A.D., revealing three different building phases. In order to investigate specific patterns and shifts through time in the use of cattle the genetic data is evaluated in relation to osteometric patterns and occurrence of pathologies on cattle metapodia. Males were genotyped for a Y-chromosomal SNP in *UTY19* that separates the two major haplogroups, Y1 and Y2, in taurine cattle. A subset of the samples were also genotyped for one SNP involved in coat coloration (*MC1R),* one SNP putatively involved in resistance to cattle plague (*TLR4)*, and one SNP in intron 5 of the *IGF-1* gene that has been associated to size and reproduction.

The results of the molecular analyses confirm that the skeletal assemblage from Eketorp is dominated by skeletal elements from females, which implies that dairying was important. Pathological lesions on the metapodia were classified into two groups; those associated with the use as draught animals and those lesions without a similar aetiology. The results show that while bulls both exhibit draught related lesions and other types of lesions, cows exhibit other types of lesions. Interestingly, a few elements from females exhibit draught related lesions. We conclude that this reflects the different use of adult female and male cattle.

Although we note some variation in the use of cattle at Eketorp between Iron Age and Medieval time we have found little evidence for the use of different types of animals for specific purposes. The use of specific (genetic) breeds seems to be a phenomenon that developed later than the Eketorp settlement.

## Introduction

Breeding of cattle has been suggested to have a long tradition in Europe [1]. For example in Italy (Etruria) some contemporary breeds are believed to predate the Roman age [2]. In Northern Europe there may be native breeds, which can be traced some 1000 years back in time [3]. Although farming was introduced to Scandinavia during the Neolithic it is from the Bronze Age and onwards that livestock was used more regularly in the cultivation of land [4] The interaction between farming and animal husbandry became more expressed during the Iron Age [4]–[7].

**Figure 1:**
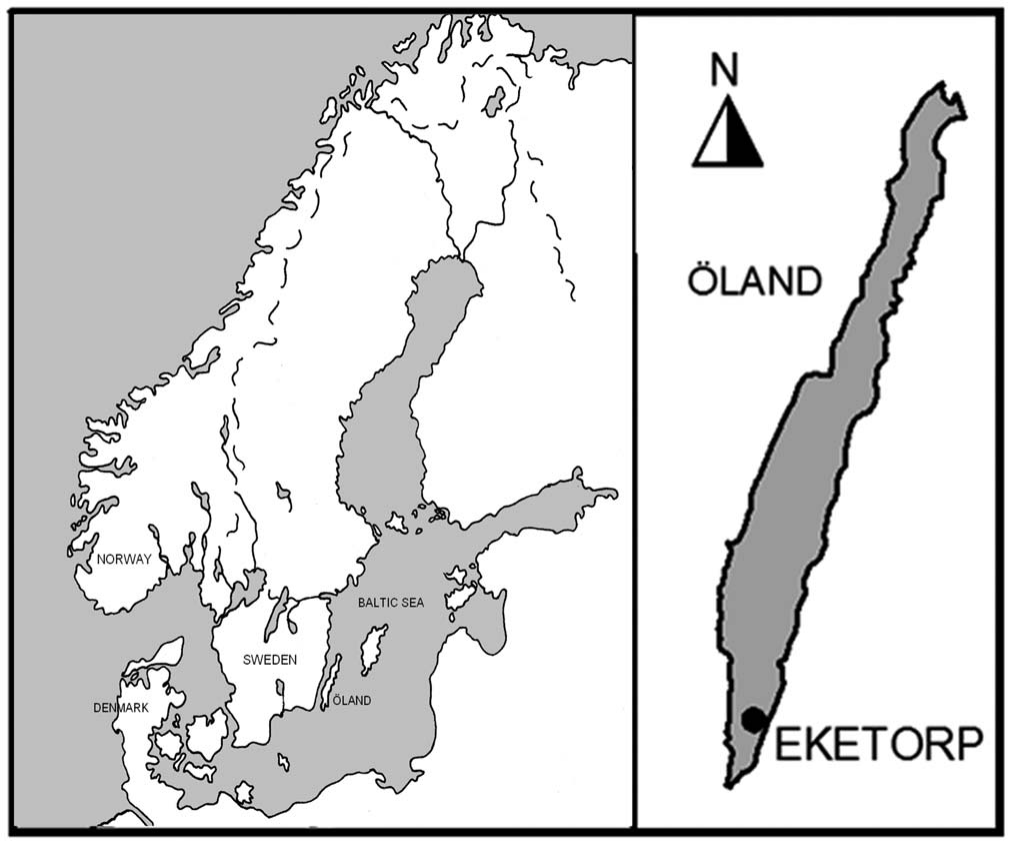
Map showing the location of Öland and Eketorp.

During the Viking Age and Medieval Age there were various trade markets in the Baltic region, organized through several ports. The ports also served as hubs for the spread of new agricultural technological innovations and often farms were located close to these ports. The Eketorp ringfort, in the southern parts of Öland, Sweden (Figure 1) offers a unique insight into Iron Age and medieval period husbandry. At an early stage the ringfort was a farming settlement, which over time developed to a garrison. Three settlement phases, I-III, have been identified. Phase I is from Late Roman Iron Age ca. 300–400 A.D. and phase II from Germanic Iron Age ca.400–700 A.D. The ringfort was then abandoned and used again from about 1170–1220 A.D. [8], [9]. The animal bones from the ringfort have previously been studied by Boessneck et al (1979). The vast majority of the osteological material is from the second and third phases of the settlement, and thus we have focused on these two periods.

Some of the 53 house foundations dating to phase II at Eketorp are remains of three-aisled houses [10] which were a new type that were built with a byre for stalling animals during winter [11]–[13]. The remains of 13 byres with stalls for approximately one hundred cattle have been excavated [14]. The faunal assemblage recovered at the Eketorp ringfort is one of the largest in Scandinavia from that time period; 0.5 tons from phase II and 1.3 tons from phase III [8], [15]. The recovered bones represent food debris from domesticates such as cattle, sheep and pig, where cattle probably was the most important source of meat. Approximately 75% of the slaughtered adult animals were females [15], [16], which illustrate the frequency bias in sex when females are kept to an adult age for milk production. In phase III the bones mainly represent debris of meat that had been brought to the site. Stables have been identified but they do not contain byres for cattle. The presence of long and slender metapodia shows that castration was practiced during both phases (II and III) [15], [16]. Fifty-seven bones (23,1%) out of a total of 247 metatarsals and 41 bones (15,2%) of 269 metacarpals exhibit pathological lesions.

The mortality pattern for cattle shows a dominance of bones from sub adult animals in both phases. This is confirmed by Boessneck et al's data on tooth eruption and epiphysial closure on tibia and metapodials where 53.6% in phase II and 59.3% in phase III of the cattle were slaughtered before the age of 2 ½ years old. Boessneck et al also presents a size comparison of unfused first phalanx showing that the majority of sub adult cattle were slaughtered between 6–8 month and 1 ½ – 1 ¾ year of age. The calves were mainly slaughtered in the late autumn and early winter [15]. The average withers height for Eketorp bulls was 111 cm and for cows 109 cm in phase II while it in phase III was approximately two cm higher for both sexes. Compared to cattle from other Swedish and North European sites the Eketorp cattle from period III are among the largest [15], [17].

Body size has been used to a wide extent to discuss prehistoric breeding and changes in body size have often been seen as a sign of improvement of livestock [18].Biomolecular analyses may provide complimentary data to the osteological data and holds the possibility to identify genetically different types of animals. Variation in genes coding for specific traits, such as, pigmentation, and muscle mass, may be used to detect breeding [19]–[21]. However, as breed identification of modern animals demands a panel of some 30 microsatellite markers [22] an even larger panel of SNPs suitable for degraded DNA would be needed [23]. This makes genetic breed identification based on ancient DNA (aDNA) a challenging task given the poor preservation of the DNA and the techniques available at present. We choose to investigate size differences that may be related to genetic characteristics, and if such exist, it would be an indication of advanced animal husbandry. Also, changes in single genetic systems over time can be an indication of selection/breeding.

Here we use a combination of morphological data; sex, physical characteristics and pathological patterns (studied by Telldahl) with molecular data on sex and genetic variation to identify possible shifts in cattle breeding strategies at Eketorp. We consider Eketorp as a model site for northern European farming. If this is a key period in rapid sophisticated specialisation within farming, we expect to find a change in sex proportions and morphology. If, on the other hand, we do not find such change, it can be taken as support for continuity, or a slower rate of specialisation during this period.

## Materials and Methods

Our study focuses on metapodia from cattle (*Bos taurus*) recovered at Eketorp. A total of 4470 metapodia have been identified at Eketorp whereof 1879 are from phase II and 2572 from phase III [15]. The bones are highly fragmented and from the assemblage we were able to retrieve 340 specimens of metapodia that offered possibilities for osteometric analyses and/or analyses of specific skeletal lesions. The total sample comprises of 190 metatarsalia and 150 metacarpalia (McIII-IV and Mt III–IV – hereafter abbreviated Mc or Mt). Both complete and fragmentary metapodia from fully-grown and sub adult cattle were analysed. In cattle the distal epiphysis in metacarpals fuses at the age of 2–2 ½ and metatarsals fuses approximately ½ years later [24]–[26]. In order to investigate the slaughter patterns of calves at Eketorp bones from sub adults were also selected for molecular sexing.

For size comparisons we use the breadth of the distal epiphysis (Bd) taken according to definitions from von den Driesch [27]. This measurement has proven useful for morphological sexing of male and female metapodia [28], [29] and is commonly used in osteoarchaeological analyses. All measurements were documented using a digital calliper to the nearest 0.01 mm.

Seven different types of skeletal lesion on metapodia were identified in the Eketorp assemblage by Telldahl: lipping, new bone formation, eburnation, bone inflammation causing thickening of the diaphysis, depressions in articular facet, carpals/tarsals ankylosis and broadening of trochlea capitis medialis of metapodia. Lipping and broadening of trochlea capitis of metapodia is an overgrowth or bone modification beyond the joint margins. Exostosis is seen as new bone formation near the articular facets. Eburnation is seen as polished bone surface where the cartilage is damaged [30]. Thickening of the diaphysis could be the result of an inflammation where bacteria has access through the connective tissue[31]. The depressions are recorded in both proximal and distal articular facets. The etiology of carpal/tarsal ankylosis is uncertain but research have shown a correlation with age, conformation of the legs and increased load [32].

**Figure 2:**
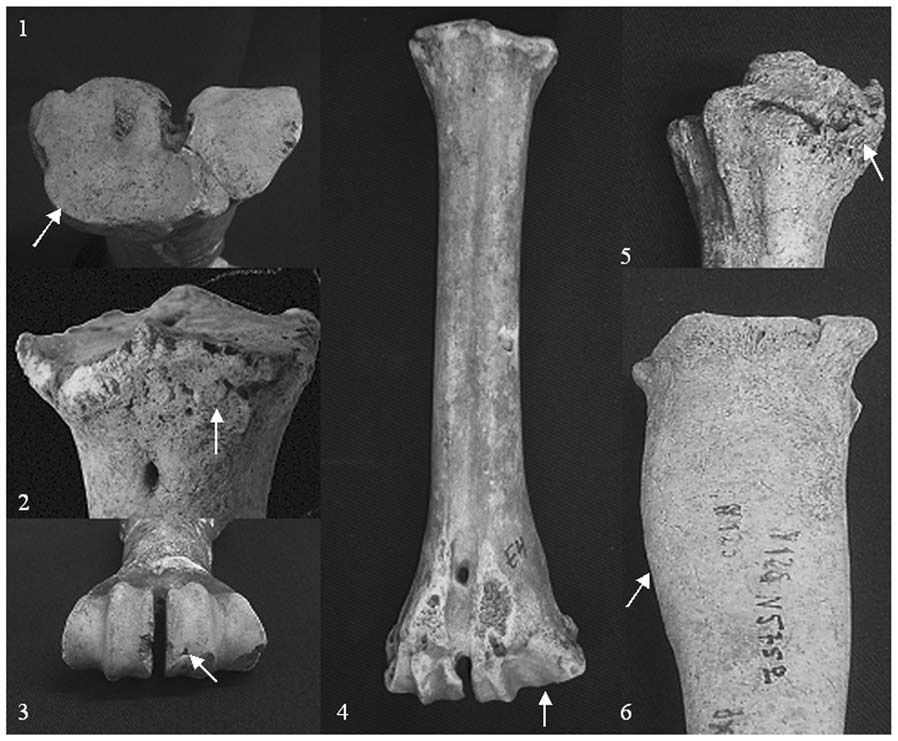
Six skeletal lesions in metapodials from Eketorp ringfort, Öland, Sweden. The lesions comprise of lipping (1), exostosis (2), depression on distal trochlea (3), broadening of the medial trochlea (4), tarsal ankylosis (5) and bone inflammation causing thickening of the diaphysis (6).

Two of these lesions, lipping and broadening of trochlea capitis medialis, are probably related to the use as draught animals [33], [34], [49] (Figure 2). In the present study the lesions are classified into two groups, lesions associated with draught use (workload related) and lesions with an unknown aetiology, i.e. not with certainty related to draught use. Here we report the lesions as present or absent.

A total of 133 metapodia dated to phase II and III were chosen for molecular analyses; 44 metacarpals and 89 metatarsals including 31 metatarsals from juvenile animals with an unfused distal epiphysis too young to be sexed morphometrically; 13 from phase II and 18 from phase III. The selection of the bones was conducted in order to gain a representative sample covering as completely as possible the full morphological size variation and also the presence of skeletal pathologies. A laboratory dedicated to work on aDNA, physically separated from work on modern DNA and PCR products, with positive air pressure and UV lightning was used for all aDNA extractions, a previously described method [21] was used and one extraction blank was included per every six extracts. PCR for sex identification was set up as in [28] using the forward sequencing system (F+R−biotinylated PCR primers and forward sequencing primer) S4 targeting a 63bp fragment. Positive PCR products were genotyped using pyrosequencing technology with a PSQ 96MA following guidelines from the manufacturer.

All samples identified as males were further genotyped for a Y chromosomal SNP in *UTY19,* this SNP has been shown to differentiate North and South European breeds in modern cattle, haplogroup Y1 and Y2 respectively [35]. A primer set targeting 74 base pairs was developed to increase the amplification success of the relatively degraded DNA (Figure S4). A subset of the samples genotyped for the sex identifying SNP were selected for further genotyping based on molecular preservation. Samples from both periods were genotyped in one SNP located in intron 5 of the *Insulin-like Growth Factor 1* (*IGF-1*) gene [38] (Figure S4). *IGF-1* is essential for in vivo follicular development in cattle [39], [40] and it is also known to play an important role in various aspects of muscle growth and development [41], [42]. The SNP is located in a QTL for twinning rate [38], [43], and has been picked up in genome scans for selection in modern cattle [38], [44], [45]. Further one SNP involved in coat coloration in the *Melanocortin receptor 1* (*MC1R)* gene [36] and one SNP putatively involved in resistance to cattle plague, *Toll-like receptor 4* (*TLR4)* [37] were genotyped. These two SNPs have been suggested to be under selection in northern European cattle (REF 21, Svensson et al, 2007 animal genetics) and the same PCR and sequencing conditions as in (21) was used.

All new primer systems were first blasted to ensure specificity to cattle, and tested on human DNA in the optimization process. The nature of pyrosequencing, where not only the SNP position, but also adjacent nucleotides is given also ensures that correct and specific results are obtained. Allelic dropout was assumed in cases where one or more replicates were homozygous while the other replicates were heterozygous or homozygous for another allele. An estimate of the probability of a false homozygote after (n) replicates was calculated according to [46]: P (false homozygote)  =  K x (K/2)^n-1^, where K is the observed number of allelic dropouts divided by all heterozygous individuals. Allele frequencies were calculated by hand. Chi 2 test and Fisher's exact test as implemented in STATISTICA 9 were used to test for differentiation in allele frequencies between period II and III. Detailed descriptions information of each element is provided in the supporting information (Figures S1, S2, S3).

## Results

DNA was successfully extracted from 104 of the 133 metapodia chosen for analysis, based on a minimum of 4 typings for females and 2 typings for males. However, 4 samples yielded insufficient data for a conclusive result. We were unable to extract DNA from 28 of the bones. The success rate was 78.9 % (Table 1). Allelic dropout was 0.33, providing significance with 4 observations for a female (p false homozygote = 0.001476). No bias was detected in which of the two alleles that was lost in male samples with allelic dropout. The osteological sex identification was confirmed by the molecular result in all cases.

**Table 1:** Results of molecular sexing.

Adults	Phase	Female	Male	no result
Metacarpals	II	3	3	2
	III	11	14	10
	II/III			1
**Total**		**14**	**17**	**13**
Metatarsals	II	6	11	4
	III	16	15	4
	II/III	2	1	2
**Total**		**24**	**27**	**10**
Subadult metatarsals				
	II	2	7	3
	III	8	5	3
**Total**		**10**	**12**	**6**

**Figure 3:**
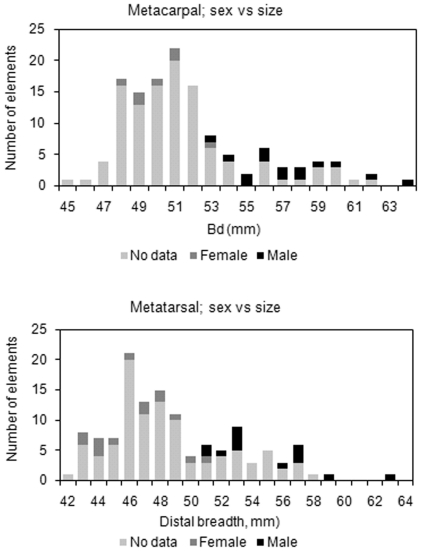
Histograms showing the size of cattle metatarsals and metatarsals according to the breadth of the distal epiphysis (Bd). Sex according to molecular analyses. Included are all available measurements.

The size distribution confirms that female animals dominate the Eketorp assemblage in both phase II and III. The distal breadth of the epiphysis of the metacarpal and metatarsal bone shows a good separation of the sexes with a small overlap around 53 mm for Mc and around 51 mm for Mt (Figure 3). Mean values and standard deviations of the distal breadth on successfully sexed metapodials confirm a minor overlap (Table 2). DNA analysis of young animals indicate that, more males were slaughtered at young age in period II compared to period III, however the differences is not significant, p = 0.09 (Chi2) (Table 1).

**Table 2:** Results of the mean value and standard deviation (s.d.) of the distal epiphysis (Bd) in molecular sexed metapodials from Eketorp ringfort, Öland in Sweden.

		Female			Male		
Adults	Phase	n	mean	s.d.	n	mean	s.d.
Metacarpals	II	2	52.61	1.52	3	57.53	1.27
	III	5	49.72	1.19	11	58.58	3.31
Metatarsals	II	2	45.28	2.79	4	57.06	2.50
	III	10	46.67	2.48	8	54.81	4.25

**Figure 4:**
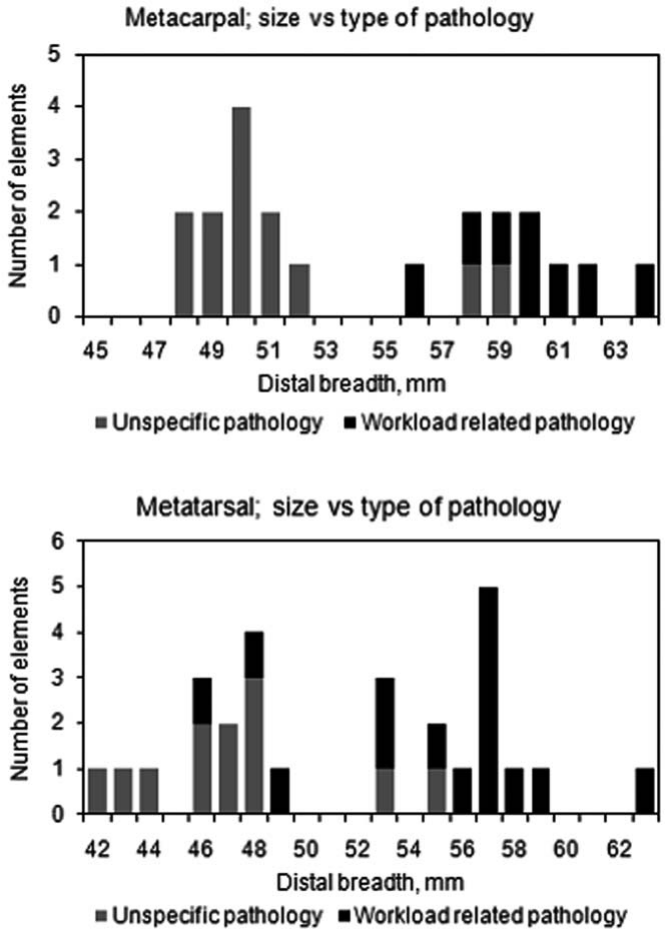
Histograms showing the size variation of metapodia with unspecific or workload related pathological lesions.

The number of different fully grown elements identified was 135 metatarsals and 151 metacarpals. Twenty-three of the metatarsals elements exhibited pathological lesions of an unspecific aetiology while 13 elements (9.6%) exhibited draught related lesions. For metacarpals the corresponding frequencies were 20 unspecific and 8 (5.3%) draught related lesions. There is a slight difference in frequency of draught lesions between Mc and Mt in period II while the frequencies are more even in period III, the observations are too few for a more detailed interpretation, Table 3. The lesions associated to draught use were found mainly on metapodia from male animals while the other types of lesions are found on bones mainly coming from female animals (Figure 4). Noteworthy in Phase III is that three metatarsals exhibiting workload related pathologies apparently come from female cattle on the basis of their distal breath measurement. Molecular sexing was inconclusive for two of the elements while the third one was not analysed (Figure S3).

**Table 3:** Frequency of pathological lesions on cattle metapodia at Eketorp in phase II and III studied by molecular analyses.

		Female		Male		No result	
Element	Phase	Unspecificpat.	Workload related	Unspecific pat.	Workload related	Unspecificpat.	Workload related
Metacarpals	II	2		1	1	4	3
	III	10		4	4		
Metatarsals	II	3		3	5	1	
	III	8	1	7	3		2
	II/III	1			1		1

**Figure 5:**
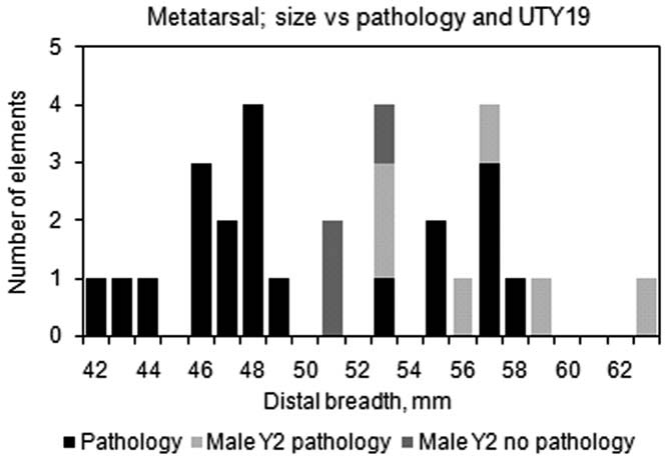
Histogram showing size variation and pathological lesions for males correlated with Y haplogroup.

**Figure 6:**
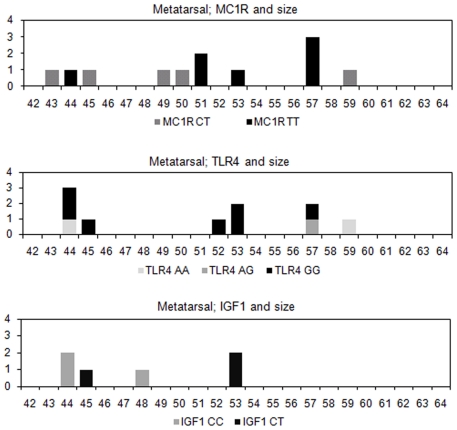
Histograms showing the size variation of the genotyped animals (metatarsals) for the *MC1R*, *TLR4* and *IGF1* genes.

Thirty-three male metapodia were successfully typed for the *UTY19* SNP, all but two have the Y2 defining allele (A). One sub adult from period II and one adult from period III belong to haplogroup Y1(C). The metapodia of the Y2 males exhibit a marked size variation (Figure 5). Only a limited number of animals were genotyped for the coat colour SNP *MC1R* (n = 34), but the result is interesting; no animals indicated the C/C genotype consistent with dominant black coat colour. Instead all animals were either heterozygous (which also results in black pigmentation) or homozygous for the wild type allele, which suggests that the Eketorp cattle mainly were of red or light coat coloration. The *MC1R* results did not yield any significant difference between the two periods, possibly due to limited sample size, or because there was no difference in the coat coloration. When 85 animals were genotyped for the 2021C>T *TLR4* SNP putatively involved in resistance to cattle plague, no significant change in allele frequency over time p = 0.08 was found (Fishers exact two-tailed). The ancestral G allele (G is due to reverse sequencing of the SNP), linked to possible resistance to cattle plague that increases from 0.605 in period II to 0.769 in period III. Finally, 18 animals were typed for a C/T mutation in *IGF-1*, five from Eketorp II and 13 from Eketorp III. The difference between the two periods is on the verge of significant (p = 0.0532) indicating an increase of allele C, from 0.4 to 0.77, in phase III, but it should be noted that the sample set is relatively small. No obvious morphological trait could be assigned to the variation in *IGF-1*. However it should be noted that the frequency of the C allele in modern milk and meat breeds is 0.9 and 0.71 respectively, thus the allele frequency in Eketorp III is more similar to modern beef cattle. No size clusters correlated to *IGF-1* genotype are visible, since all genotyped animals fell within the same size range as the other animals (Figure 6).

## Discussion

Our results confirm that the skeletal assemblage of cattle at Eketorp mainly consists of bones from female animals. The predominance of cows is by no means unusual as cows provided both milk and calves for breeding purposes. The need for bulls was not as great and only a handful was most likely enough in order to cover the need for breeding. The use of cattle was not restricted to dairying or meat procurement. At Eketorp the metapodia of males and females exhibit a different pattern of pathological lesions. Females exhibit a dominance of lesions with an unspecific etiology while males also exhibited lesions that may be associated to draught use.

**Figure 7:**
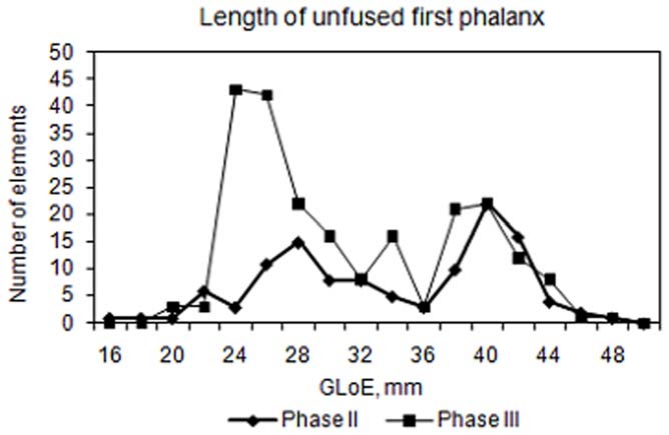
Line graph showing the size variation of unfused first phalanx at Eketorp, phases II and III. The GloE24-30 mm represent calves aged between 6–8 month and GloE 38–43 mm represents calves between 1 ½–1 ¾ years of age. Data are found in Boessneck (et al. 1979:tab 21).

**Figure 8:**
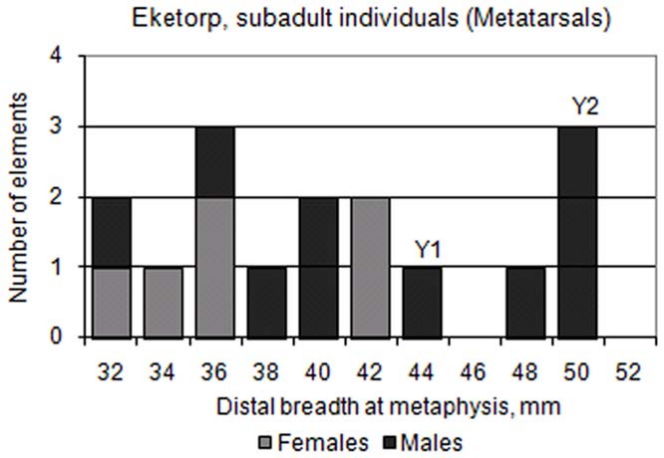
Histogram showing the size distribution of the sub adult metatarsals in phase III.

Boessneck [15] observed that calves at Eketorp exhibited a seasonal pattern of slaughter (Figure 7). Phase II exhibits a roughly similar frequency of calves slaughtered in their first or second autumn while phase III shows a clear dominance of calves slaughtered in their first autumn (*ibid.*). The comparison is to some extent affected by the inclusion of both anterior and posterior elements, which may exhibit a slightly different growth pattern. This however is of no major consequence for the comparison. Furthermore, female and male cattle exhibit a different growth pattern. We show that the majority of the sub adult cattle were males, which is in accordance with the (opposite) sex distribution of the adults (Figure 8).

The difference in kill-off patterns between phases II and III is related to an increased reliance on meat, which is in line with the change of the ringfort from a farming settlement to a fortified complex. However, the kill off pattern in phase III was not an ideal one towards husbandry practices focusing on milk production. Instead it indicates a specialization for meat production to the ringfort. The culling of many female calves is not commonly observed and, in fact, this may have led to a depletion of the cattle stock around Eketorp. Linked to this may have been the need to use females for draught purposes during phase III. Earlier studies [15], [16] show that castration was most probably conducted on some male animals at Eketorp and thus a link between castration and draught use may be assumed. Systematic breeding of draught cattle is known in written sources in Sweden since the 16^th^ Century when it became a profitable trade, especially in Southern Sweden when farming expanded [47]. We have no indications that the castrated males have been of a selected breed or specific type of animal. Bulls may have been chosen from a common pool of animals. All studied castrates belonged to haplogroup Y2, but note that we only observed two cases of Y1 among all males analysed.

The increase in body size observed by Boessneck et al. [15] might imply an introduction of a different type of livestock during phase III. However, we found no support for the use of different types of animals for specific purposes. The genetic data indicate that the cattle population at Eketorp was homogeneous both within each phase and between phase II and III, the latter also indicates that the population of cattle on Öland wasn't subjected to any major genetic changes for at least 400 years. There is no statistically significant difference in genetic data, but trends indicate possible differences. Taken together, the genetic result and the morphology suggest that there is a small but noticeable difference between the population from period II and that from period III. The resistance to cattle plague was possibly slightly higher in phase III. Only in less than one case out of ten would we expect to see the present difference *TLR4* by pure chance, the trend is even more obvious in *IGF-1*. If the trends are interpreted as true differences, then the animals were probably exposed to natural or artificial selection that is disease or breeding. However, given the time elapsed between the two periods and the relatively small sample set genetic drift could also explain the differences in allele frequencies.

Because of the relatively isolated location, the ringfort was probably dependent on local cattle. The military ringfort during phase III probably had to have some kind of organization to secure meat resources. But, it is unlikely that large numbers of cattle of different breeds were imported to Eketorp from other areas. However, it cannot be excluded that some animals or meat from animals that did not belong to the (local) breeding population were brought to the ringfort. Two of the male animals belong to a different Y-chromosomal haplogroup (Y1) than the majority of males, which similarly to other animals from early medieval northern Europe belong to haplogroup Y2 [48]. Although, this SNP is correlated with breeds in modern animals [35], we cannot claim that this is the case for the Eketorp cattle; since analysis of aDNA have shown temporal rather than geographical structure in historic populations [28]. It is therefore more appropriate to state that a minimum of two different male lineages were present at the site during both periods, one in majority and one very rare.

Summarizing our results, we uncovered trends that can be interpreted as a chronological shift, possibly towards a farming economy where cattle gained a new role. We also discovered patterns, compatible with early breeding and an increased level of specialisation in phase III, although these patterns were not obvious enough to be interpreted as undisputable evidence. We see a varied use of cattle at Eketorp and its surroundings, utilization strategies that require breeding efforts and conscious decisions on actions such as castration. In spite of this we have found little if any evidence of the use of genetically specific types of animals for specific purposes. The use of specific (genetic) breeds seems to be a later phenomenon. The usage of the bulk of the cattle seems to have been constant from period II to period III, indicating that the shift we describe was not a fast one.

### Ethics statement

The animal bones used in this article are food debris from the excavated Eketorp ringfort on the Öland island in Sweden dated between 300–1200/50 A.D. The Museum of National Antiquities, Stockholm, Sweden, has permitted the analysis.

## Supporting Information

Figure S1 **Descriptive data on metatarsals including DNA results.**
(DOC)

Figure S2 **Descriptive data on metacarpals including DNA results.**
(DOC)

Figure S3 **Information on genotypic data, including number of successful genotypes for each SNP.**
(DOC)

Figure S4 **Primers and input data for the pyrosequencing software for PCR amplification and genotyping of** ***IGF1, UTY19 and ZFX/Y*** **.**
(DOC)
