# Supplementary material for: Typing Late Prehistoric Cows and Bulls—Osteology and Genetics of Cattle at the Eketorp Ringfort on the Öland Island in Sweden
Source: PLoS One. 2011 Jun 22;6(6):e20748. doi: 10.1371/journal.pone.0020748 (PMC3120812; doi:10.1371/journal.pone.0020748)
Supplement: Figure S1 — Descriptive data on metatarsals including DNA results. (DOC) [file pone.0020748.s001.doc]

S1. Descriptive data on metatarsals including DNA results.

| **Element** | **ID** | **Age** | **DNA NBR** | **Phase** | **Sex DNA** | **Pathology** |  | **Work related pat. (yes/no)** | **TLR4** | **IGF1** | **UTY19 Y1/2** | **MC1R** | **Side sin/dx)** | **Bd (mm)** |
| --- | --- | --- | --- | --- | --- | --- | --- | --- | --- | --- | --- | --- | --- | --- |
| Metatarsal | X80 |  | 21 | III | Female | n |  | n |  |  |  | CT | s | 49,58 |
| Metatarsal | Y81 |  |  | II |  | n |  | n |  |  |  |  | d | 46,5 |
| Metatarsal | X81 |  |  | III |  | n |  | n |  |  |  |  | s | 46,82 |
| Metatarsal | X81 |  |  | III |  | y |  | y |  |  |  |  | d | 54,01 |
| Metatarsal | Y99 |  |  | II |  | y |  | n |  |  |  |  | d |  |
| Metatarsal | X100 |  |  | III |  | n |  | n |  |  |  |  | d | 48,03 |
| Metatarsal | X104 |  | E32 | III | Male | y |  | y |  |  | 2 |  | d | 56,22 |
| Metatarsal | X104 |  |  | III |  | n |  | n |  |  |  |  | d | 43,48 |
| Metatarsal | X/Y119 |  | E28 | II/III | Male | y |  | y |  | CT | 2 |  | d | 53,03 |
| Metatarsal | X120 | Sub ad | eu3 | III | Female |  |  |  |  |  |  |  |  |  |
| Metatarsal | X121 |  |  | III |  | y |  | y |  |  |  |  |  | 52,25 |
| Metatarsal | X/Y123 |  |  | II/III |  | n |  | n |  |  |  |  | s | 53,97 |
| Metatarsal | Y125 |  | E6 | II | Male | y |  | y | GG | CT |  | TT | d | 53,56 |
| Metatarsal | Y125 | Sub ad | eu22 | II |  |  |  |  |  |  |  |  |  |  |
| Metatarsal | Y127 |  |  | II |  | n |  | n |  |  |  |  | d | 49,17 |
| Metatarsal | X128 |  |  | III |  | n |  | n |  |  |  |  | d | 56,01 |
| Metatarsal | X141 |  |  | III |  | n |  | n |  |  |  |  | d | 46,6 |
| Metatarsal | X143 |  |  | III |  | y |  | n |  |  |  |  | s | 55,44 |
| Metatarsal | X/Y144 |  | E26 | II/III | Female | y |  | n |  |  |  |  | s | 48,5 |
| Metatarsal | X145 |  |  | III |  | n |  | n |  |  |  |  |  | 50,52 |
| Metatarsal | X146 |  | E82 | III | Female | n |  | n | GG | CC |  |  | d | 44,59 |
| Metatarsal | Y147 |  |  | II |  | n |  | n |  |  |  |  | d | 46,12 |
| Metatarsal | Y148 |  | E24 | II | Female | y |  | n |  |  |  |  | d | 47,25 |
| Metatarsal | X148 |  |  | III |  | n |  | n |  |  |  |  | d | 46,35 |
| Metatarsal | X149 | Sub ad | eu12 | III | Female |  |  |  |  |  |  |  |  | 36,67 |
| Metatarsal | Y150 |  | E35 | II |  | n |  | n |  |  |  |  | s | 43,72 |
| Metatarsal | X151 |  | E83 | III | Female | n |  | n |  |  |  |  | d | 46,9 |
| Metatarsal | X152 |  |  | III |  | y |  | y |  |  |  |  | s | 48,43 |
| Metatarsal | Y162 |  |  | II |  | n |  | n |  |  |  |  | d | 46,71 |
| Metatarsal | Y167 |  |  | II |  | y |  | n |  |  |  |  | d |  |
| Metatarsal | Y168 |  | 15 | II | Female | n |  | n |  |  |  | CT | d | 43,31 |
| Metatarsal | X168 |  | E29 | III | Male | y |  | n |  |  | 2 |  | d |  |
| Metatarsal | X172 |  |  | III |  | n |  | n |  |  |  |  | d | 53,67 |
| Metatarsal | X174 |  | E34 | III |  | y |  | y |  |  |  |  | d | 46,85 |
| Metatarsal | X177 |  |  | III |  | n |  | n |  |  |  |  | d | 55,48 |
| Metatarsal | X189 |  |  | III |  | n |  | n |  |  |  |  | s | 52,18 |
| Metatarsal | X191 |  |  | III |  | n |  | n |  |  |  |  | d | 52,3 |
| Metatarsal | Y192 | Sub ad | eu20 | II | Male |  |  |  |  |  |  |  |  | 41,06 |
| Metatarsal | X192 |  |  | III |  | y |  | y |  |  |  |  | s | 57,67 |
| Metatarsal | X194 |  | E50 | III | Male | y |  | n | GG |  | 2 |  | s | 53,1 |
| Metatarsal | Y197 |  |  | II |  | n |  | n |  |  |  |  | s | 49,87 |
| Metatarsal | X/Y198 |  | 19 | II/III | Female | n |  | n |  |  |  | CT | d | 50,81 |
| Metatarsal | X198 |  |  | III |  | n |  | n |  |  |  |  | d | 46,33 |
| Metatarsal | X199 |  | E79 | III | Female | y |  | n | GG |  |  | TT | s | 44,91 |
| Metatarsal | Y199 |  | E2 | II | Male | y |  | y | AA |  | 2 | CT | d | 59,46 |
| Metatarsal | X200 | Sub ad | E80 | III | Female |  |  |  | GG |  |  | TT | s |  |
| Metatarsal | Y200 |  |  | II |  | n |  | n |  |  |  |  | s | 48,5 |
| Metatarsal | X202 | Sub ad | eu14 | III | Female |  |  |  |  |  |  |  |  |  |
| Metatarsal | Y202 |  | E1 | II | Male | y |  | n | AG | TT | 2 | CT | d |  |
| Metatarsal | X202 |  |  | III |  | n |  | n |  |  |  |  | s | 47,18 |
| Metatarsal | X213 |  | E12 | III | Male | y |  | n | AG |  | 1 |  | s |  |
| Metatarsal | X213 |  |  | III |  | n |  | n |  |  |  |  | d | 46,67 |
| Metatarsal | X214 |  | 18 | III | Male | n |  | n |  |  | 2 | TT | s | 51,77 |
| Metatarsal | X215 |  | E15 | III | Male | y |  | n |  |  |  |  | d |  |
| Metatarsal | X215 |  |  | III |  | n |  | n |  |  |  |  | d | 53,72 |
| Metatarsal | X217 |  | E38 | III |  | y |  | y |  |  |  |  | d | 58,3 |
| Metatarsal | Y217 |  | E31 | II | Male | y |  | n |  |  | 2 |  | s |  |
| Metatarsal | X217 |  |  | III |  | n |  | n |  |  |  |  | d | 48,32 |
| Metatarsal | X217 |  |  | III |  | n |  | n |  |  |  |  | d | 54,76 |
| Metatarsal | X/Y220 |  |  | II/III |  | n |  | n |  |  |  |  | s | 49,6 |
| Metatarsal | X222 |  | E27 | III | Female | y |  | n |  |  |  |  | d |  |
| Metatarsal | X/Y224 |  |  | II/III |  | y |  | y |  |  |  |  | s | 55,25 |
| Metatarsal | X225 |  | E76 | III | Female | n |  | n |  |  |  |  | s | 47,56 |
| Metatarsal | X225 | Sub ad | eu5 | III | Male |  |  |  |  |  |  |  |  | 51,53 |
| Metatarsal | X225 |  |  | III |  | n |  | n |  |  |  |  | s | 45,3 |
| Metatarsal | X226 |  |  | III |  | y |  | n |  |  |  |  | d | 47,09 |
| Metatarsal | X240 |  |  | III |  | n |  | n |  |  |  |  | d | 46,13 |
| Metatarsal | X241 |  |  | III |  | n |  | n |  |  |  |  | s | 44,47 |
| Metatarsal | Y245 |  | E5 | II | Male | y |  | y | GG |  |  | TT | s | 57,85 |
| Metatarsal | X247 |  |  | III |  | n |  | n |  |  |  |  | d | 44,55 |
| Metatarsal | X250 |  | E20 | III | Female | y |  | n |  | CC |  |  | d | 48,29 |
| Metatarsal | Y251 | Sub ad | eu19 | II | Male |  |  |  |  |  |  |  |  | 51,13 |
| Metatarsal | Y251 |  | E43 | II | Male | n |  | n | AG |  |  |  | s |  |
| Metatarsal | X252 |  |  | III |  | n |  | n |  |  |  |  | d | 46,43 |
| Metatarsal | X252 | Sub ad | eu4 | III |  |  |  |  |  |  |  |  |  | 49,65 |
| Metatarsal | X253 |  |  | III |  | n |  | n |  |  |  |  | d | 43,62 |
| Metatarsal | X253 |  |  | III |  | n |  | n |  |  |  |  | d | 49,16 |
| Metatarsal | X263 |  | E4 | III | Male | y |  | y |  |  | 2 |  | d | 63,97 |
| Metatarsal | X264 |  | 22 | III |  | n |  | n |  |  |  |  | d | 49,07 |
| Metatarsal | X265 |  |  | III |  | n |  | n |  |  |  |  | d | 46,06 |
| Metatarsal | X267 |  |  | III |  | y |  | n |  |  |  |  | d |  |
| Metatarsal | X269 |  |  | II/III |  | n |  | n |  |  |  |  | d | 51,78 |
| Metatarsal | X/Y274 |  | E77 | III | Female | n |  | n | GG | CT |  | CT | d | 45,29 |
| Metatarsal | X274 |  |  | III |  | y |  | n |  |  |  |  | s | 46,34 |
| Metatarsal | X276 | Sub ad | eu1 | III | Female |  |  |  |  |  |  |  |  | 43,2 |
| Metatarsal | X276 |  | E22 | III | Female | y |  | n |  |  |  |  | s |  |
| Metatarsal | X276 |  |  | III |  | n |  | n |  |  |  |  | s | 46,38 |
| Metatarsal | X277 |  |  | III |  | n |  | n |  |  |  |  | d | 48,47 |
| Metatarsal | X279 |  |  | III |  | n |  | n |  |  |  |  | d | 51,65 |
| Metatarsal | X287 |  |  | III |  | n |  | n |  |  |  |  | d | 44,98 |
| Metatarsal | Y288 | Sub ad | eu24 | II | Male |  |  |  |  |  |  |  |  |  |
| Metatarsal | X289 | Sub ad | eu13 | III | Female |  |  |  |  |  |  |  |  | 33,28 |
| Metatarsal | X289 |  |  | III |  | y |  | n |  |  |  |  | d |  |
| Metatarsal | Y290 |  | E36 | II | Male | n |  | n |  |  |  |  | s |  |
| Metatarsal | X291 |  | E78 | III | Male | n |  | n | AG |  | 2 |  |  |  |
| Metatarsal | X/Y292 |  |  | II/III |  | n |  | n |  |  |  |  |  | 50 |
| Metatarsal | X293 |  |  | III |  | n |  | n |  |  |  |  | s | 44,3 |
| Metatarsal | X/Y294 |  | E30 | II/III |  | y |  | y |  |  |  |  | d | 57,18 |
| Metatarsal | X/Y297 |  |  | II/III |  | n |  | n |  |  |  |  | d | 47,27 |
| Metatarsal | X/Y297 |  |  | II/III |  | n |  | n |  |  |  |  | s | 48,95 |
| Metatarsal | X/Y300 |  |  | II/III |  | n |  | n |  |  |  |  | s | 45,61 |
| Metatarsal | X301 |  |  | III |  | n |  | n |  |  |  |  | s | 48,02 |
| Metatarsal | X302 |  |  | III |  | n |  | n |  |  |  |  | d | 46,08 |
| Metatarsal | X303 | Sub ad | eu8 | III | Male |  |  |  |  |  |  |  |  | 38,32 |
| Metatarsal | X317 |  |  | III |  | n |  | n |  |  |  |  | s | 46,07 |
| Metatarsal | X317 |  |  | III |  | y |  | y |  |  |  |  | s | 55,05 |
| Metatarsal | X/Y322 |  |  | II/III |  | n |  | n |  |  |  |  | s | 47,11 |
| Metatarsal | X/Y323 |  |  | II/III |  | n |  | n |  |  |  |  | d | 49,92 |
| Metatarsal | X325 |  |  | III |  | n |  | n |  |  |  |  | s | 46,11 |
| Metatarsal | X326 |  |  | III |  | n |  | n |  |  |  |  | d | 52,44 |
| Metatarsal | Y327 |  | E10 | II | Male | y |  | y | AG |  | 2 | TT | s | 57,03 |
| Metatarsal | X327 |  | E17 | III | Male | y |  | n |  |  |  |  | s |  |
| Metatarsal | X327 |  |  | III |  | n |  | n |  |  |  |  | s | 45,41 |
| Metatarsal | X327 |  |  | III |  | n |  | n |  |  |  |  | s | 45,77 |
| Metatarsal | X327 |  |  | III |  | n |  | n |  |  |  |  | d | 49,27 |
| Metatarsal | X341 |  |  | III |  | n |  | n |  |  |  |  | d | 45,11 |
| Metatarsal | X342 |  | E81 | III | Male | n |  | n |  |  | 2 |  | d | 53,09 |
| Metatarsal | X342 |  |  | III |  | n |  | n |  |  |  |  | s | 43,9 |
| Metatarsal | X344 | Sub ad | eu10 | III | Male |  |  |  |  |  |  |  |  | 33,08 |
| Metatarsal | X/Y348 |  | 14 | II/III |  | n |  | n |  |  |  |  | d | 51,18 |
| Metatarsal | X352 |  | E3 | III | Male | y |  | n |  |  | 2 |  | d |  |
| Metatarsal | Y356 |  | E39 | II | Female | n |  | n | AG |  |  |  | s |  |
| Metatarsal | Y357 | Sub ad | eu17 | II | Male |  |  |  |  |  |  |  |  | 45,51 |
| Metatarsal | X363 |  | E18 | III | Female | y |  | n |  |  |  |  | s |  |
| Metatarsal | X364 |  |  | III |  | n |  | n |  |  |  |  | d | 49,84 |
| Metatarsal | Y365 |  | E40 | II |  | n |  | n |  |  |  |  | s |  |
| Metatarsal | X366 |  |  | III |  | n |  | n |  |  |  |  | d | 43,32 |
| Metatarsal | X367 |  |  | III |  | n |  | n |  |  |  |  | d | 56,15 |
| Metatarsal | X/Y369 |  |  | II/III |  | n |  | n |  |  |  |  | s | 48,43 |
| Metatarsal | X370 |  | E7 | III | Female? | y |  | y |  |  |  |  | s | 49,99 |
| Metatarsal | Y370 | Sub ad | eu27 | II | Male |  |  |  |  |  |  |  |  | 41,1 |
| Metatarsal | Y371 |  |  | II |  | n |  | n |  |  |  |  | d | 47,21 |
| Metatarsal | X374 |  | 5 | III | Female | n |  | n |  |  |  |  | d | 51,29 |
| Metatarsal | Y374 |  |  | II |  | y |  | n |  |  |  |  | d |  |
| Metatarsal | Y375 |  |  | II |  | n |  | n |  |  |  |  | d | 46,14 |
| Metatarsal | X379 |  |  | III |  | n |  | n |  |  |  |  | d | 47,99 |
| Metatarsal | Y390 |  | E42 | II |  | n |  | n |  |  |  |  | s |  |
| Metatarsal | X390 |  |  | III |  | y |  | n |  |  |  |  | s |  |
| Metatarsal | Y392 | Sub ad | eu28 | II |  |  |  |  |  |  |  |  |  |  |
| Metatarsal | X394 |  | E9 | III | Male | y |  | n | AA |  |  |  | d |  |
| Metatarsal | X395 | Sub ad | eu11 | III |  |  |  |  |  |  |  |  |  | 38,54 |
| Metatarsal | X396 |  | E75 | III | Male | y |  | y | GG | CC |  |  | d |  |
| Metatarsal | X396 |  |  | III |  | n |  | n |  |  |  |  | d | 52,88 |
| Metatarsal | X397 |  | E73 | III | Female | n |  | n | AA | CC |  | TT | d | 44,41 |
| Metatarsal | X398 | Sub ad | E8 | III | Female? |  |  |  | GG | CT |  |  | d |  |
| Metatarsal | X398 | Sub ad | eu16 | III |  |  |  |  |  |  |  |  |  |  |
| Metatarsal | Y399 |  | E37 | II | Male | n |  | n | AG |  | 2 |  | s |  |
| Metatarsal | X400 | Sub ad | eu6 | III | Male |  |  |  |  |  |  |  |  | 51,97 |
| Metatarsal | X400 |  |  | III |  | n |  | n |  |  |  |  | d | 48,16 |
| Metatarsal | X414 |  |  | III |  | y |  | y |  |  |  |  | d | 57,17 |
| Metatarsal | X416 |  |  | III |  | y |  | n |  |  |  |  | d | 42,56 |
| Metatarsal | Y422 |  | E41 | II | Female | n |  | n | AA | CT |  | CT | s |  |
| Metatarsal | X423 |  | E74 | III | Male | n |  | n | GG |  |  |  | d | 52,31 |
| Metatarsal | X424 |  |  | III |  | n |  | n |  |  |  |  | d | 46,52 |
| Metatarsal | X436 | Sub ad | eu2 | III | Male |  |  |  |  |  |  |  |  | 48,78 |
| Metatarsal | Y440 | Sub ad | eu23 | II | Male |  |  |  |  |  |  |  |  |  |
| Metatarsal | X442 |  |  | III |  | n |  | n |  |  |  |  | d | 55,91 |
| Metatarsal | Y443 |  |  | II |  | n |  | n |  |  |  |  | s | 48 |
| Metatarsal | X443 |  |  | III |  | n |  | n |  |  |  |  | d | 54,57 |
| Metatarsal | X444 |  |  | III |  | y |  | n |  |  |  |  | d | 46,11 |
| Metatarsal | X460 |  | E33 | III | Female | y |  | n |  |  |  |  | s | 43,92 |
| Metatarsal | X461 |  |  | III |  | n |  | n |  |  |  |  | d | 49,6 |
| Metatarsal | X464 |  |  | III |  | n |  | n |  |  |  |  | d | 43,9 |
| Metatarsal | Y465 |  | 6 | II | Male | y |  | y |  |  | 2 | TT | d | 57,38 |
| Metatarsal | X480 |  |  | III |  | n |  | n |  |  |  |  | d | 47,84 |
| Metatarsal | X/Y482 |  |  | II/III |  | n |  | n |  |  |  |  | s | 53,4 |
| Metatarsal | X/Y1d |  |  | II/III |  | n |  | n |  |  |  |  | d | 48,25 |
| Metatarsal | X151b |  |  | III |  | n |  | n |  |  |  |  | s | 48,14 |
| Metatarsal | X151c |  |  | III |  | n |  | n |  |  |  |  | s | 47,31 |
| Metatarsal | X1a | Sub ad | eu15 | III | Female |  |  |  |  |  |  |  |  |  |
| Metatarsal | Y1b | Sub ad | eu25 | II | Male |  |  |  |  |  |  |  |  | 36,86 |
| Metatarsal | X200b | Sub ad | eu7 | III | Female |  |  |  |  |  |  |  |  | 43,46 |
| Metatarsal | X200b |  |  | III |  | n |  | n |  |  |  |  | s | 47,8 |
| Metatarsal | X202b | Sub ad | eu9 | III | Female |  |  |  |  |  |  |  |  | 37,04 |
| Metatarsal | Y317b | Sub ad | E19 | II | Female |  |  |  | AG |  |  |  | d |  |
| Metatarsal | X328b |  |  | III |  | y |  | n |  |  |  |  | s | 48,95 |
| Metatarsal | X397a |  | 17 | III | Male | n |  | n |  |  | 2 | TT | d | 51,01 |
| Metatarsal | X397b |  |  | III |  | y |  | n |  |  |  |  | d |  |
| Metatarsal | X81b |  |  | III |  | n |  | n |  |  |  |  | d | 45,76 |
| Metatarsal | YH21 |  |  | II |  | n |  | n |  |  |  |  | d | 47,25 |
| Metatarsal | XH3 |  | 20 | III |  | n |  | n |  |  |  |  | d | 50,03 |
| Metatarsal | YH5 |  |  | II |  | y |  | n |  |  |  |  | d |  |
| Metatarsal | YH5a |  | E16 | II | Male | y |  | n |  |  |  |  | d |  |
| Metatarsal | YH6 | Sub ad | eu18 | II |  |  |  |  |  |  |  |  |  |  |
| Metatarsal | YH6b | Sub ad | eu21 | II | Female |  |  |  |  |  |  |  |  |  |
| Metatarsal | YHB | Sub ad | eu26 | II | Female |  |  |  |  |  |  |  |  | 35,44 |
| Metatarsal | Y1c |  | E11 | II | Female | y |  | n | GG |  |  |  | s |  |
| Metatarsal | XSP |  |  | III |  | n |  | n |  |  |  |  | s | 53,92 |
| Metatarsal | Y1e |  |  | II |  | n |  | n |  |  |  |  | d | 47,96 |
| Metatarsal | YWT |  | E13 | II |  | y |  | n |  |  |  |  | s |  |
