# Supplementary material for: Typing Late Prehistoric Cows and Bulls—Osteology and Genetics of Cattle at the Eketorp Ringfort on the Öland Island in Sweden
Source: PLoS One. 2011 Jun 22;6(6):e20748. doi: 10.1371/journal.pone.0020748 (PMC3120812; doi:10.1371/journal.pone.0020748)
Supplement: Figure S2 — Descriptive data on metacarpals including DNA results. (DOC) [file pone.0020748.s002.doc]

S2. Descriptive data on metacarpals including DNA results.

| **Element** | ID | **Age** | **DNA NBR** | **Phase** | **Sex DNA** | **Pathology (yes/no)** | **Workrelated pat (Yes/no)** | **TLR4** | **IGF1** | **UTY19 Y1/2** | **MC1R** | **Side (sin/dx)** | **Bd** |
| --- | --- | --- | --- | --- | --- | --- | --- | --- | --- | --- | --- | --- | --- |
| Metacarpal | X2 |  |  | III |  | n | n |  |  |  |  | s | 47,98 |
| Metacarpal | X14 |  |  | III |  | y | n |  |  |  |  | d | 50,34 |
| Metacarpal | X/Y79 |  |  | II/III |  | y | n |  |  |  |  | - | 50,17 |
| Metacarpal | X80 |  |  | III |  | n | n |  |  |  |  | d | 51,45 |
| Metacarpal | X81 |  | 11 | III | Male | n | n |  |  | 2 |  | d | 57,27 |
| Metacarpal | X81 |  |  | III |  | n | n |  |  |  |  | s | 49,06 |
| Metacarpal | X99 |  |  | III |  | n | n |  |  |  |  | s | 51,11 |
| Metacarpal | X/Y101a |  |  | II/III |  | n | n |  |  |  |  | d | 45,79 |
| Metacarpal | X/Y101b |  |  | II/III |  | n | n |  |  |  |  | d | 49,54 |
| Metacarpal | X101 |  |  | III |  | n | n |  |  |  |  | s | 52,42 |
| Metacarpal | X104 |  |  | III |  | n | n |  |  |  |  | s | 51,3 |
| Metacarpal | X120 |  | E68 | III | Female | y | n |  |  |  |  | s | 51,3 |
| Metacarpal | Y124 |  | E51 | II | Female | y | n | GG |  |  | TT | d | 51,54 |
| Metacarpal | X124 |  |  | III |  | n | n |  |  |  |  | s | 48,59 |
| Metacarpal | X124 |  |  | III |  | n | n |  |  |  |  | d | 50,19 |
| Metacarpal | X126 |  |  | III |  | n | n |  |  |  |  | s | 51,56 |
| Metacarpal | X141 |  | E49 | III | Female | y | n | GG |  |  |  | d | 50,3 |
| Metacarpal | X/Y142 |  |  | II/III |  | n | n |  |  |  |  | d | 52,37 |
| Metacarpal | Y143 |  | E14 | II | Male | y | y |  |  |  |  | s | 56,79 |
| Metacarpal | X/Y145 |  |  | II/III |  | n | n |  |  |  |  | d | 51,14 |
| Metacarpal | X145 |  |  | III |  | n | n |  |  |  |  | d | 51,34 |
| Metacarpal | X146 |  | E63 | III | Female | y | n |  |  |  |  | s |  |
| Metacarpal | X147 |  |  | III |  | n | n |  |  |  |  | s | 50 |
| Metacarpal | Y150a |  |  | II |  | n | n |  |  |  |  | s | 50,73 |
| Metacarpal | Y150b |  |  | II |  | n | n |  |  |  |  | d | 51,63 |
| Metacarpal | X151a |  |  | III |  | n | n |  |  |  |  | s | 48,64 |
| Metacarpal | X151b |  |  | III |  | n | n |  |  |  |  | s | 56,75 |
| Metacarpal | X165 |  | E23 | III | Male | y | n | AG | CT | 2 |  | d | 59,91 |
| Metacarpal | X165 |  | E70 | III | Male | y | y | GG | CC | 2 | TT | d | 62,61 |
| Metacarpal | Y165 |  |  | II |  | n | n |  |  |  |  | s | 50,35 |
| Metacarpal | Y166 |  | E61 | II | Male | y | n | GG | CT |  |  | d |  |
| Metacarpal | X/Y167 |  |  | II/III |  | y | n |  |  |  |  | s | 48,37 |
| Metacarpal | Y168 |  |  | II |  | n | n |  |  |  |  | d | 47,44 |
| Metacarpal | X168a |  |  | III |  | n | n |  |  |  |  | s | 48,37 |
| Metacarpal | X/Y168 |  |  | II/III |  | y | n |  |  |  |  | d | 48,93 |
| Metacarpal | X168b |  |  | III |  | n | n |  |  |  |  | s | 59,69 |
| Metacarpal | X169 |  | E46 | III | Female | n | n |  |  |  |  | d | 49,36 |
| Metacarpal | X171 |  | 3 | III |  | n | n |  |  |  |  | d | 57,54 |
| Metacarpal | X172 |  |  | III |  | n | n |  |  |  |  | s | 52,58 |
| Metacarpal | X173 |  |  | III |  | n | n |  |  |  |  | d | 49,91 |
| Metacarpal | X173 |  |  | III |  | n | n |  |  |  |  | d | 51,44 |
| Metacarpal | X174 |  | E59 | III |  | y | y |  |  |  |  | s | 60,13 |
| Metacarpal | Y174a |  |  | II |  | n | n |  |  |  |  | d | 51,06 |
| Metacarpal | X174b |  |  | III |  | n | n |  |  |  |  | d | 52,32 |
| Metacarpal | X174c |  |  | III |  | n | n |  |  |  |  | s | 52,65 |
| Metacarpal | X174d |  |  | III |  | n | n |  |  |  |  | d | 52,7 |
| Metacarpal | Y174e |  |  | II |  | y | n |  |  |  |  | s |  |
| Metacarpal | X189 |  |  | III |  | n | n |  |  |  |  | d | 54,23 |
| Metacarpal | X191 |  |  | III |  | n | n |  |  |  |  | s | 50,63 |
| Metacarpal | X191 |  |  | III |  | n | n |  |  |  |  | d |  |
| Metacarpal | X192 |  | E57 | III |  | y | n |  |  |  |  | s | 58,77 |
| Metacarpal | Y193 |  | 16 | II |  | n | n |  |  |  |  | s | 56,09 |
| Metacarpal | X194 |  | E47 | III |  | y | n |  |  |  |  | d | 49,27 |
| Metacarpal | X196 |  | E66 | III | Female | y | n | AG | CT |  | TT | s |  |
| Metacarpal | X196 |  |  | III |  | n | n |  |  |  |  | s | 49,79 |
| Metacarpal | X196 |  |  | III |  | n | n |  |  |  |  | d | 60,21 |
| Metacarpal | X201 |  |  | III |  | n | n |  |  |  |  | d | 47,33 |
| Metacarpal | Y203 |  |  | II |  | y | n |  |  |  |  | d |  |
| Metacarpal | X/Y212 |  |  | II/III |  | n | n |  |  |  |  | d | 48,8 |
| Metacarpal | X213 |  |  | III |  | n | n |  |  |  |  | s | 49,77 |
| Metacarpal | X213 |  |  | III |  | n | n |  |  |  |  | s | 50,95 |
| Metacarpal | X214 |  | E56 | III | Male | y | y | GG |  | 2 |  | s | 64,76 |
| Metacarpal | X215 |  |  | III |  | n | n |  |  |  |  | s | 48,89 |
| Metacarpal | X217 |  |  | III |  | n | n |  |  |  |  | d | 48,44 |
| Metacarpal | X/Y220 |  |  | II/III |  | n | n |  |  |  |  | d | 53,85 |
| Metacarpal | X225 |  |  | III |  | n | n |  |  |  |  | d | 51,21 |
| Metacarpal | X226 |  | E69 | III | Female | y | n | GG |  |  |  | s |  |
| Metacarpal | Y227 |  | 4 | II | Male | n | n |  |  | 2 |  | d | 56,8 |
| Metacarpal | X247 |  |  | III |  | n | n |  |  |  |  | d | 52,05 |
| Metacarpal | Y250 |  |  | II |  | n | n |  |  |  |  | d | 49,93 |
| Metacarpal | X250 |  |  | III |  | n | n |  |  |  |  | d | 52,29 |
| Metacarpal | X251 |  | E54 | III |  | n | n |  |  |  |  | d | 62,42 |
| Metacarpal | Y251 |  |  | II |  | n | n |  |  |  |  | d | 50,1 |
| Metacarpal | X251 |  |  | III |  | n | n |  |  |  |  | d | 51,56 |
| Metacarpal | X252 |  | E58 | III |  | y | y |  |  |  |  | s | 61,39 |
| Metacarpal | X252 |  | E21 | III | Male | y | n |  | CT |  |  | d |  |
| Metacarpal | X252 |  | E64 | III | Male | y | n |  |  |  |  | s |  |
| Metacarpal | X252 |  |  | III |  | n | n |  |  |  |  | s | 49,57 |
| Metacarpal | X252 |  |  | III |  | n | n |  |  |  |  | d | 50,74 |
| Metacarpal | X263 |  |  | III |  | n | n |  |  |  |  | d | 52,01 |
| Metacarpal | X265 |  |  | III |  | n | n |  |  |  |  | d | 49,99 |
| Metacarpal | X265 |  |  | III |  | n | n |  |  |  |  | d |  |
| Metacarpal | X267 |  |  | III |  | n | n |  |  |  |  | s | 50,15 |
| Metacarpal | X/Y268 |  |  | II/III |  | n | n |  |  |  |  | s | 51,91 |
| Metacarpal | X/Y268 |  |  | II/III |  | n | n |  |  |  |  | s | 53,59 |
| Metacarpal | X271 |  | 12 | III | Male | n | n |  |  | 2 | TT | s | 57,28 |
| Metacarpal | X273 |  |  | III |  | n | n |  |  |  |  | d | 52,76 |
| Metacarpal | Y274 |  |  | II |  | y | n |  |  |  |  | d | 52,79 |
| Metacarpal | X289 |  |  | III |  | n | n |  |  |  |  | s | 51,51 |
| Metacarpal | X291 |  |  | III |  | n | n |  |  |  |  | d | 50,58 |
| Metacarpal | X293 |  |  | III |  | n | n |  |  |  |  | s | 51,11 |
| Metacarpal | X/Y294 |  |  | II/III |  | n | n |  |  |  |  | d | 48,5 |
| Metacarpal | X295 |  |  | III |  | n | n |  |  |  |  | d | 52,73 |
| Metacarpal | X/Y300 |  |  | II/III |  | n | n |  |  |  |  | s | 52,72 |
| Metacarpal | Y301 |  |  | II |  | n | n |  |  |  |  | s | 52,03 |
| Metacarpal | X301 |  |  | III |  | n | n |  |  |  |  | d | 54,51 |
| Metacarpal | X302 |  | E65 | III | Male | y | n |  |  | 2 |  | s |  |
| Metacarpal | X315 |  | 8 | III | Male? | n | n |  |  |  | CT | d | 54,68 |
| Metacarpal | X/Y319 |  |  | II/III |  | n | n |  |  |  |  | d | 50,76 |
| Metacarpal | X321 |  | E71 | III |  | y | y |  |  |  |  | s | 59,2 |
| Metacarpal | X/Y321 |  |  | II/III |  | n | n |  |  |  |  | s | 49,27 |
| Metacarpal | X/Y324 |  |  | II/III |  | n | n |  |  |  |  | s | 48,89 |
| Metacarpal | X326 |  | E25 | III |  | y | n |  |  |  |  | d |  |
| Metacarpal | X326 |  | E53 | III | Female | y | n | GG |  |  |  | s |  |
| Metacarpal | X327 |  | 10 | III | Male | n | n |  |  | 2 |  | s | 55 |
| Metacarpal | Y342 |  | E72 | II | Female | n | n | AA | CT |  | TT | d | 53,69 |
| Metacarpal | Y343 |  |  | II |  | n | n |  |  |  |  | s | 51,94 |
| Metacarpal | X349 |  | E48 | III | Female | y | n | AA |  |  |  | d |  |
| Metacarpal | X349 |  |  | III |  | y | n |  |  |  |  | s | 50,36 |
| Metacarpal | X352 |  | 9 | III | Male | n | n |  |  | 2 | CT | s | 53,47 |
| Metacarpal | X362 |  | E44 | III | Female | y | n | AG | CC |  | TT | d | 49,57 |
| Metacarpal | X367 |  | E60 | III | Male | y | y |  |  | 2 |  | s | 60,46 |
| Metacarpal | X368 |  | E67 | III |  | y | n |  |  |  |  | s |  |
| Metacarpal | X368 |  | E55 | III | Male | y | y | GG |  |  |  | d | 58,99 |
| Metacarpal | Y368 |  |  | II |  | n | n |  |  |  |  | - | 48,98 |
| Metacarpal | Y373 |  |  | II |  | n | n |  |  |  |  | d | 51,62 |
| Metacarpal | X373 |  |  | III |  | n | n |  |  |  |  | s | 52,45 |
| Metacarpal | X/Y390 |  |  | II/III |  | n | n |  |  |  |  | d | 51,08 |
| Metacarpal | X393 |  |  | III |  | n | n |  |  |  |  | s | 48,85 |
| Metacarpal | X394 |  | E45 | III | Female | y | n | AA | CC |  |  | d | 48,07 |
| Metacarpal | X395 |  |  | III |  | n | n |  |  |  |  | s | 53,79 |
| Metacarpal | X396 |  | 7 | III | Male | n | n |  |  | 2 | TT | s | 58,79 |
| Metacarpal | X397 |  |  | III |  | n | n |  |  |  |  | s | 48,48 |
| Metacarpal | X398 |  | 13 | III | Male | n | n |  |  | 2 |  | s | 55,88 |
| Metacarpal | X399 |  |  | III |  | n | n |  |  |  |  | d | 54,45 |
| Metacarpal | X412 |  |  | III |  | n | n |  |  |  |  | s | 53,12 |
| Metacarpal | X422 |  |  | III |  | n | n |  |  |  |  | d | 50,04 |
| Metacarpal | X438 |  |  | III |  | n | n |  |  |  |  | - | 53,45 |
| Metacarpal | X441 |  |  | III |  | n | n |  |  |  |  | d | 52,06 |
| Metacarpal | Y442 |  |  | II |  | n | n |  |  |  |  | d | 46,26 |
| Metacarpal | X465 |  | E52 | III | Female | y | n | GG |  |  |  | s |  |
| Metacarpal | X616 |  |  | III |  | n | n |  |  |  |  | s | 56,82 |
| Metacarpal | X/Y2882 |  | 1 | II/III |  | n | n |  |  |  |  | s | 56,96 |
| Metacarpal | X1i |  |  | III |  | n | n |  |  |  |  | s | 48,23 |
| Metacarpal | X1j |  |  | III |  | n | n |  |  |  |  | s | 48,34 |
| Metacarpal | X1k |  |  | III |  | n | n |  |  |  |  | s | 49,56 |
| Metacarpal | Y1h |  |  | II |  | y | n |  |  |  |  | s | 51,14 |
| Metacarpal | Y1l |  |  | II |  | n | n |  |  |  |  | d | 51,57 |
| Metacarpal | X1m |  |  | III |  | n | n |  |  |  |  | d | 51,96 |
| Metacarpal | Y1n |  |  | II |  | n | n |  |  |  |  | - | 53,38 |
| Metacarpal | X1o |  |  | III |  | n | n |  |  |  |  | s | 54,59 |
| Metacarpal | X1g |  |  | III |  | n | n |  |  |  |  | d | 50,06 |
| Metacarpal | X169b |  |  | III |  | n | n |  |  |  |  | d | 47,78 |
| Metacarpal | X/Y318 |  |  | II/III |  | n | n |  |  |  |  | d | 49,74 |
| Metacarpal | X/Y321b |  |  | II/III |  | n | n |  |  |  |  | - | 49,81 |
| Metacarpal | YH3 |  |  | II |  | y | n |  |  |  |  | d |  |
| Metacarpal | YH5 |  | E62 | II | Female | y | n |  |  |  |  | s |  |
| Metacarpal | YH5 |  |  | II |  | n | n |  |  |  |  | s | 48,71 |
| Metacarpal | X/Y1f |  |  | II/III |  | n | n |  |  |  |  | - | 59,99 |
| Metacarpal | YWT |  | 2 | II |  | n | n |  |  |  |  | s | 60,48 |
