# Supplementary material for: Typing Late Prehistoric Cows and Bulls—Osteology and Genetics of Cattle at the Eketorp Ringfort on the Öland Island in Sweden
Source: PLoS One. 2011 Jun 22;6(6):e20748. doi: 10.1371/journal.pone.0020748 (PMC3120812; doi:10.1371/journal.pone.0020748)
Supplement: Figure S3 — Information on genotypic data, including number of successful genotypes for each SNP. (DOC) [file pone.0020748.s003.doc]

S3. Information on genotypic data, including number of successful genotypes for each SNP.

| **Hmid** | **FindID** | **DNA ID** | ***ZFX/Y*** | **Typings** | ***UTY19*** | **Typings** | ***TLR4*** | **Typings** | ***IGF1*** | **Typings** | ***MC1R*** | **Typings** |
| --- | --- | --- | --- | --- | --- | --- | --- | --- | --- | --- | --- | --- |
|  |  |  |  |  |  |  |  |  |  |  |  |  |
| 2255:4 | Y202 | E1 | C/T | 4 | A | 5 | A/G | 6 | T/T | 4 | C/T | 2 |
| 7255:4 | Y199 | E2 | C/T | 7 | A | 2 | A/A | 12 |  |  | C/T | 2 |
| 2280:1 | X352 | E3 | C/T | 4 | A | 2 |  | 2 |  |  |  |  |
| 2280:1 | X263 | E4 | C/T | 4 | A | 3 |  | 3 |  |  |  |  |
| 7287:8 | Y245 | E5 | C/T | 7 |  |  | G/G | 6 |  |  | T/T | 2 |
| 7255:4 | Y125 | E6 | C/T | 5 |  |  | G/G | 6 | C/T | 2 | T/T | 2 |
| 7287:8 | X370 | E7 | T/T | 7 |  |  |  | 1 |  |  |  |  |
| 2271:9 | X398 | E8 | T/T | 10 |  |  | G/G | 4 | C/T | 4 |  |  |
| 2280:1 | X394 | E9 | C/T | 5 |  |  |  |  |  |  |  |  |
| 7252:17 | Y327 | E10 | C/T | 3 | A | 2 | A/G | 10 |  |  | T/T | 4 |
| 7252:14 | Y1c | E11 | T/T | 7 |  |  | G/G | 4 |  |  |  | 1 |
| 2271:1 | X213 | E12 | C/T | 7 | C | 3 | A/G | 2 |  |  |  |  |
| 7252:17 | Y143 | E14 | C/T | 8 |  |  |  | 1 |  |  |  |  |
| 2280:1 | X215 | E15 | C/T | 6 |  |  |  |  |  |  |  |  |
| 2255.4 | Y115 | E16 | C/T | 5 |  |  |  |  |  |  |  |  |
| 2271:7 | X327 | E17 | C/T | 5 |  |  |  |  |  |  |  |  |
| 2271:7 | X363 | E18 | T/T | 4 |  |  |  |  |  |  |  |  |
| 7255:5 | Y317b | E19 | T/T | 6 |  |  | A/G | 3 |  |  |  |  |
| 2280:2 | X250 | E20 | T/T | 8 |  |  |  |  | C/C | 4 |  |  |
| 7251:3 | X252 | E21 | C/T | 5 |  |  |  |  | C/T | 4 |  |  |
| 2271:7 | X276 | E22 | T/T | 7 |  |  |  |  |  |  |  |  |
| 7255:7 | X165 | E23 | C/T | 6 | A | 3 | A/G | 4 | C/T | 4 |  |  |
| 7255:5 | Y148 | E24 | T/T | 6 |  |  |  | 3 |  |  |  |  |
| 7287:5 | XY144 | E26 | T/T | 6 |  |  |  |  |  |  |  |  |
| 2271:9 | X222 | E27 | T/T | 9 |  |  |  |  |  |  |  |  |
| 2271:14 | XY119 | E28 | C/T | 3 | A | 3 |  |  | C/T | 3 |  |  |
| 2271:1 | X168 | E29 | C/T | 4 | A | 7 |  |  |  |  |  |  |
| 2280:1 | X217 | E31 | C/T | 4 | A | 1 |  |  |  |  |  |  |
| 2280:1 | X104 | E32 | C/T | 7 | A | 2 |  |  |  |  |  |  |
| 2280:2 | X460 | E33 | T/T | 7 |  |  |  |  |  |  |  |  |
| 7252:14 | Y290 | E36 | C/T | 4 |  |  |  |  |  |  |  |  |
| 7252:14 | Y399 | E37 | C/T | 6 |  | 6 | A/G | 9 |  |  |  | 1 |
| 7252:14 | Y356 | E39 | T/T | 6 |  |  | A/G | 3 |  |  |  |  |
| 7252:14 | Y422 | E41 | T/T | 6 |  |  | A/A | 6 | C/T | 4 | T/T | 4 |
| 7252:17 | Y251 | E43 | C/T | 5 |  |  | A/G | 6 |  |  |  |  |
| 7251:1 | X362 | E44 | T/T | 8 |  |  | A/G | 7 | C/C | 12 | T/T | 4 |
| 7251:1 | X394 | E45 | T/T | 21 |  |  | A/A | 7 | C/C | 4 |  |  |
| 7251:1 | X169 | E46 | T/T | 7 |  |  |  |  |  |  |  |  |
| 7251:1 | X349 | E48 | T/T | 7 |  |  | A/A | 4 |  |  |  |  |
| 7251:1 | X141 | E49 | T/T | 7 |  |  | G/G | 4 |  |  |  |  |
| OFL | X194 | E50 | C/T | 4 | A | 5 | G/G | 4 |  |  |  |  |
| OFL | Y124 | E51 | T/T | 6 |  |  | G/G | 6 |  |  | T/T | 8 |
| 7251:12 | X465 | E52 | T/T | 6 |  |  | G/G | 4 |  |  |  |  |
| 7251:12 | X326 | E53 | T/T | 8 |  |  | G/G | 4 |  |  |  |  |
| 7251:10 | X368 | E55 | C/T | 6 |  |  | G/G | 4 |  |  |  |  |
| 7251:8 | X214 | E56 | C/T | 4 | A | 2 | G/G | 4 |  |  |  |  |
| 7251:8 | X367 | E60 | C/T | 6 | A | 2 |  |  |  |  |  |  |
| 7251:11 | Y166 | E61 | C/T | 5 |  |  | G/G | 4 | C/T | 2 |  |  |
| 7251:11 | YH5 | E62 | T/T | 8 |  |  |  | 3 |  |  |  | 1 |
| 7251:4 | X146 | E63 | T/T | 8 |  |  |  |  |  |  |  |  |
| 7251:4 | X252 | E64 | C/T | 4 |  |  | A/G | 4 |  |  |  |  |
| 7251:4 | X302 | E65 | C/T | 4 | A | 1 |  |  |  |  |  |  |
| 7251:12 | X196 | E66 | T/T | 8 |  |  | A/G | 5 | C/T | 6 | T/T | 4 |
| 7251:12 | X120 | E68 | T/T | 9 |  |  |  | 3 |  |  |  | 1 |
| 7251:12 | X226 | E69 | T/T | 6 |  |  | G/G | 5 |  |  |  |  |
| 2234:1 | X165 | E70 | C/T | 4 | A | 4 | G/G | 7 | C/C | 6 | T/T | 4 |
| 2234:1 | Y342 | E72 | T/T | 6 |  |  | A/A | 5 | C/T | 5 | T/T | 3 |
| 2280:2 | X397 | E73 | T/T | 6 |  |  | A/A | 4 | C/C | 4 | T/T | 4 |
| 2280:1 | X422 | E74 | C/T | 6 |  |  | G/G | 7 |  |  |  | 1 |
| 2280:2 | X396 | E75 | C/T | 4 |  |  | G/G | 5 | C/C | 2 |  |  |
| 2280:2 | X225 | E76 | T/T | 6 |  |  |  |  |  |  |  |  |
| 2280:2 | X274 | E77 | T/T | 8 |  |  | G/G | 5 | C/T | 2 | C/T | 4 |
| 2271:9 | X291 | E78 | C/T | 5 | A | 2 | A/G | 5 |  |  |  |  |
| 2280:2 | X199 | E79 | T/T | 8 |  |  | G/G | 5 |  |  | T/T | 4 |
| 2271:9 | X200 | E80 | T/T | 8 |  |  | G/G | 5 |  |  | T/T | 3 |
| 2280:1 | X342 | E81 | C/T | 4 | A | 2 |  | 1 |  |  |  |  |
| 2280:2 | X146 | E82 | T/T | 16 |  |  | G/G | 4 | C/C | 2 |  |  |
| 2280:2 | X151 | E83 | T/T | 9 |  |  |  |  |  |  |  |  |
| 2280:2 | X374 | 5 | T/T | 6 |  |  |  | 3 |  |  |  | 1 |
| 7255:4 | Y465 | 6 | C/T | 6 | A | 2 |  | 3 |  |  | T/T | 2 |
| OFL | Y168 | 15 | T/T | 5 |  |  |  | 3 |  |  | C/T | 5 |
| 2280:2 | X397a | 17 | C/T | 5 | A | 2 | G/G | 5 |  |  | T/T | 2 |
| 2271:1 | X217 | 18 | C/T | 10 | A | 3 | G/G | 8 |  |  | T/T | 8 |
| 2271:13 | XY198 | 19 | T/T | 10 |  |  | G/G | 4 |  |  | C/T | 7 |
| 2271:7 | X80 | 21 | T/T | 10 |  |  |  | 9 |  |  | C/T | 2 |
| 7252:7 | Y227 | 4 | C/T | 5 | A | 1 |  | 3 |  |  |  | 1 |
| 7251:8 | X396 | 7 | C/T | 9 | A | 3 | A/G | 8 |  |  | T/T | 6 |
| 7251:1 | X315 | 8 |  | 3 |  |  | G/G | 7 |  |  | C/T | 4 |
| 7251:8 | X352 | 9 | C/T | 6 | A | 3 | G/G | 4 |  |  | C/T | 3 |
| 7251:8 | X327 | 10 | C/T | 8 | A | 1 | A/G | 5 |  |  |  |  |
| 7251:10 | X81 | 11 | C/T | 4 | A | 3 | G/G | 8 |  |  |  | 1 |
| 7251:8 | X271 | 12 | C/T | 9 | A | 2 | G/G | 8 |  |  | T/T | 6 |
| OFL | X398 | 13 | C/T | 8 | A | 4 | G/G | 8 |  |  |  | 5 |
| OFL | Y193 | 16 |  | 1 |  |  | A/G | 2 |  |  |  |  |
| 2271;9 |  | eu1 | T/T | 5 |  |  | A/G | 7 |  |  |  |  |
| 2271;9 |  | eu2 | C/T | 5 |  |  |  | 2 |  |  |  |  |
| 2271;9 |  | eu3 | T/T | 5 |  |  | A/G | 10 |  |  |  | 1 |
| 2271;9 |  | eu5 | C/T | 4 | A | 1 |  |  |  |  |  |  |
| 2271;9 |  | eu6 | C/T | 6 | A | 3 | A/G | 12 |  |  |  |  |
| 2271;9 |  | eu7 | T/T | 4 |  |  |  | 1 |  |  |  |  |
| 2271;6 |  | eu8 | C/T | 3 |  |  |  |  |  |  |  |  |
| 2271;6 |  | eu9 | T/T | 4 |  |  |  | 3 |  |  |  |  |
| 2271;6 |  | eu10 | C/T | 5 |  |  |  | 2 |  |  |  |  |
| 2271;6 |  | eu12 | T/T | 6 |  |  | A/G | 12 |  |  |  | 1 |
| 2271;6 |  | eu13 | T/T | 7 |  |  | A/G | 5 |  |  |  |  |
| 2271;6 |  | eu14 | T/T | 4 |  |  |  | 1 |  |  |  |  |
| 2271;6 |  | eu15 | T/T | 5 |  |  |  |  |  |  |  |  |
| 7255;5 |  | eu17 | C/T | 5 | C | 2 |  | 3 |  |  |  |  |
| 7255;5 |  | eu19 | C/T | 3 | A | 5 | G/G | 9 |  |  |  |  |
| 7255;5 |  | eu20 | C/T | 2 |  |  |  | 1 |  |  |  |  |
| 7255;5 |  | eu21 | T/T | 6 |  |  | G/G | 5 |  |  |  |  |
| 7255;5 |  | eu23 | C/T | 2 |  |  |  | 1 |  |  |  |  |
| 7255;5 |  | eu24 | C/T | 2 |  |  |  | 1 |  |  |  |  |
| 7255;5 |  | eu25 | C/T | 2 |  |  |  | 2 |  |  |  |  |
| 7255;5 |  | eu26 | T/T | 5 |  |  | A/G | 4 |  |  |  |  |
| 7255;5 |  | eu27 | C/T | 4 |  |  |  | 3 |  |  |  |  |
| 7255;5 |  | eu28 |  |  |  |  |  | 1 |  |  |  |  |
