# Supplementary material for: Typing Late Prehistoric Cows and Bulls—Osteology and Genetics of Cattle at the Eketorp Ringfort on the Öland Island in Sweden
Source: PLoS One. 2011 Jun 22;6(6):e20748. doi: 10.1371/journal.pone.0020748 (PMC3120812; doi:10.1371/journal.pone.0020748)
Supplement: Figure S4 — Primers and input data for the pyrosequencing software for PCR amplification and genotyping of IGF1, UTY19 and ZFX/Y . (DOC) [file pone.0020748.s004.doc]

S4. Primers and input data for the pyrosequencing software for PCR amplification and genotyping of *IGF1, UTY19 and ZFX/Y*.

|  | |  | |  | | |
| --- | --- | --- | --- | --- | --- | --- |
| SNP | Forward 5’->3’ | | Reverse 5’->3’ | | Pyrosequencing 5’->3’ | Dispensationorder |
| *IGF1* | ACATGCCCAAGGCTCAGAAG | | TTGCTACAGCTCAGCCTCATAAC | | CAGCTCAGCCTCATAAC | GTCGAGTCT |
| UTY19 | ATATGGCTTGAAGCAGTCTTGAG | | GAACGTTCAAAGTTGTTTACAAA^b^ | | TGAAGCAGTCTTGAGGA |  |
| ZFX/Y | GCGTGGAGTGTGGTAAAGGT | | GATTCGCATGTGCTTTTTGA^b^ | | TGGTAAAGGTTTTCGTCA | GCTCGATCAG |

b. Biotinylated in 5’ end.
